# Intermediate Latency-Evoked Potentials of Multimodal Cortical Vestibular Areas: Galvanic Stimulation

**DOI:** 10.3389/fneur.2017.00587

**Published:** 2017-11-03

**Authors:** Stefan Kammermeier, Arun Singh, Kai Bötzel

**Affiliations:** ^1^Klinikum der Universität München, Neurologische Klinik und Poliklinik, München, Germany; ^2^Department of Neurology, University of Iowa, Iowa, IA, United States

**Keywords:** galvanic vestibular stimulation, brain-evoked source analysis, EEG, vestibular cortex, vestibular-evoked myogenic potentials

## Abstract

**Introduction:**

Human multimodal vestibular cortical regions are bilaterally anterior insulae and posterior opercula, where characteristic vestibular-related cortical potentials were previously reported under acoustic otolith stimulation. Galvanic vestibular stimulation likely influences semicircular canals preferentially. Galvanic stimulation was compared to previously established data under acoustic stimulation.

**Methods:**

14 healthy right-handed subjects, who were also included in the previous acoustic potential study, showed normal acoustic and galvanic vestibular-evoked myogenic potentials. They received 2,000 galvanic binaural bipolar stimuli for each side during EEG recording.

**Results:**

Vestibular cortical potentials were found in all 14 subjects and in the pooled data of all subjects (“grand average”) bilaterally. Anterior insula and posterior operculum were activated exclusively under galvanic stimulation at 25, 35, 50, and 80 ms; frontal regions at 30 and 45 ms. Potentials at 70 ms in frontal regions and at 110 ms at all of the involved regions could also be recorded; these events were also found using acoustic stimulation in our previous study.

**Conclusion:**

Galvanic semicircular canal stimulation evokes specific potentials in addition to those also found with acoustic otolith stimulation in identically located regions of the vestibular cortex. Vestibular cortical regions activate differently by galvanic and acoustic input at the peripheral sensory level.

**Significance:**

Differential effects in vestibular cortical-evoked potentials may see clinical use in specific vertigo disorders.

## Highlights

– Multimodal vestibular network 3-D EEG dipoles mapped with BESA.
– Specific evoked cortical potentials 25–110 ms.
– Different potentials in semicircular versus known otolith potentials.

## Introduction

The bilateral vestibular cortical regions, including the anterior insula and posterior opercular regions, have been investigated thoroughly by anatomical, functional imaging and *in vivo* stimulation studies [reviewed in Ref. (1, 2, 3)]. They receive vestibular, proprioceptive, visual inputs, and sensory re-afferent information, defining them as higher-order multimodal regions. By means of electrophysiology, early potentials up to 20 ms after acoustic, galvanic or vibratory vestibular-related stimulation are widely known and intensely debated being related to vestibular nerve, vestibular nuclear, and/or even certain early cortical activations [“oVEMP” (4–7)]. Beyond that 20 ms range, a study by our group investigated cortical-evoked potentials by acoustic-mediated stimuli directed at the saccular membrane; a specific set of evoked potentials up to 110 ms post-stimulus and induced cortical activity in the mu, beta and gamma bands up to 150 ms could be demonstrated [(8), referred to as “our previous study”]. The current study investigates the same time range beyond 20 ms with a different, galvanic vestibular stimulus directed preferentially at the semicircular canal organs (9) rather than the previously used otolith-focused specific acoustic stimulation [(8) and references therein].

Galvanic stimulation is the application of electric currents to the body. Cathodal currents can excite neurons such as vestibular afferents; anodal currents are inhibitory (10–12). Galvanic vestibular stimulation (GVS) is the application of such currents over the mastoid bones, either bilaterally with oppositely polarized electrodes (“bipolar”) or monopolar (cathodal or anodal) against a reference positioned mostly over the C7 vertebra. It is currently believed to act mainly on the unmyelinated postsynaptic transduction site of the vestibular nerve below the hair cell basilar membrane with a preference for irregularly discharging Type-I-neurons (10–18). Depending on stimulus polarity and the push–pull interaction between the two peripheral vestibular organs (19, 20–22), bilateral bipolar GVS can imitate a natural lateral leaning of head and body to the side of the cathode (18, 23–25). Effects of GVS on saccular and utricular fibers (preferentially excited by acoustic vestibular stimulation) are considered negligible (26) and can only be isolated during very specific paradigms like simultaneous specific physical rotation of the body (9). Depending on the duration of GVS stimuli, physiological responses can be vestibular perceptions, eye movements, and/or postural reactions (11, 26–29).

Galvanic stimulation can evoke vestibular-evoked myogenic potentials (gVEMP) with a positive P13 and a negative N24 in the pre-activated sternocleidomastoid neck muscle (30–38) similar to the more well-established acoustic aVEMP. Ideal parameters have been found to be short-pulsed galvanic stimuli around 1 ms of high amplitude (35), close to the individually tolerable threshold. Reference amplitudes for galvanic gVEMP have so far not been established, unlike the relatively established >100 μA normal range in acoustic aVEMP. Normal ipsilateral galvanic gVEMPs are mostly described around 20–50 µA, often together with a contralateral myogenic potential.

This study investigated whether galvanic stimulation capable of evoking neck gVEMP could also elicit specific cortical vestibular potentials, believed to resemble activity in multimodal vestibular cortical regions.

## Materials and Methods

### Subjects

Fourteen healthy right-handed individuals (seven males, seven females; age 25–32 years, average 27 years) without any history of vestibular, acoustic, or other neurological disorders were recruited from university personnel, all of whom had already participated in our previous study on acoustic cortical vestibular potentials. All had been shown to have acoustic aVEMP and specific multimodal otolith-related vestibular cortical potentials. Their Edinburgh handedness inventory scored at least 90% toward right predominance; none had history of re-education from left-handedness (two of 14 performed one of ten tasks preferentially with the left hand, all others were 100% right handed).

The setup of this study was approved by the university’s ethics committee (Decision 142/04 of the Ethikkommission der Medizinischen Fakultät der Ludwig-Maximilians-Universität). All of the involved subjects gave their written informed consent in accordance with the Declaration of Helsinki. All data were pseudonominized by study enlistment date.

### Galvanic Vestibular Stimulation

**Figure 1:**
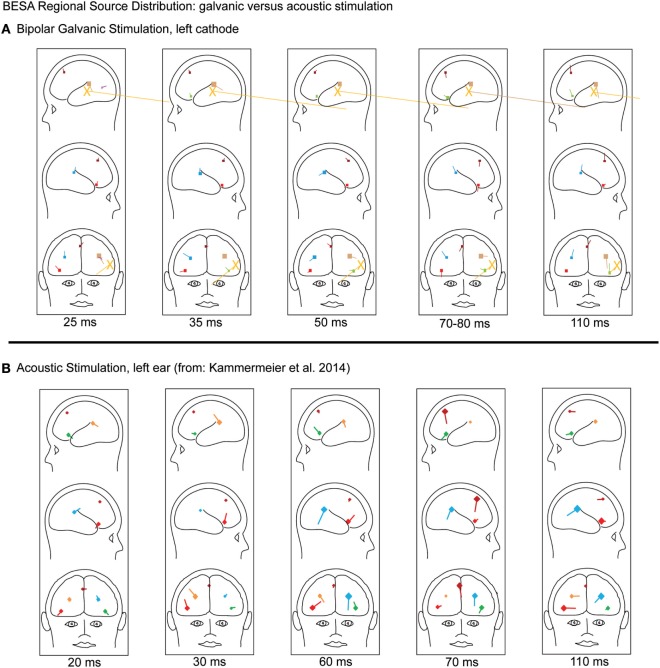
**(A)** Evoked cortical potentials in five regional sources (frontal source FV dark red; right anterior insula light red, left anterior insula light green; right posterior operculum light blue, left posterior operculum brown) of grand averaged data of 14 patients during left-sided bipolar galvanic vestibular stimulation in discrete source analysis brain-evoked source analysis. The orange large dipoles marked with X represent the combined bilateral bipolar capacitative effects of the galvanic pulse removed with principal component analysis. **(B)** Comparison to regional source dipoles evoked by acoustic vestibular stimulation in our previous study, referenced the first time as (8) in 2014. They were directed differently even in those potentials shown to have equivalent potential latency and location (70 ms in FV and 110 ms in all shown sources), indicating different activated cortical patches in the respective functional regions.

Galvanic vestibular stimulation was delivered by a Digitimer DS5 Isolated Bipolar Constant Current Stimulator, certified for clinical use (Digitimer Ltd., Hertfordshire, UK, www.digitimer.com), and connected by Ag/AgCl ring electrodes (Brain Products GmbH, Gilching, Germany) to both mastoid bones of the subject. The skin under the electrodes was prepared with a mild abrasive and filled with chloride-free electrolyte gel to minimize capacitive effects interfering with signal acquisition. Capacitive effects of the head plus the stimulus electrodes at the given short stimulus cycle times had proven particularly disturbing for signal quality in proof-of-concept trials; after optimization by Cl-free electrode gel and signal post-processing methods (specified below), the remainder of this artifact can be seen in the long-lasting and large principal components in Figure 1. Particularly long-termed GVS in the range of 300 ms pulses proved to be neither tolerable at the required intensities and repetitions nor suitable for analysis due to overlapping and intense capacitative effects.

Stimulus timing and amplitude was controlled by a galvanically detached National Instruments USB-6229 digital-analog-converter (National Instruments Germany GmbH, Munich, Germany, www.ni.com/de/), which delivered stimulus-synchronous trigger signals to the EEG recording setup. The USB-6229 output was controlled by MatLab (MatLab R2009b, The MathWorks, Natick, MA, USA, www.matlab.com).

Bilateral bipolar stimulation was chosen to imitate a near-natural vestibular cue without leaving one of the vestibular organs uninfluenced. “Side of stimulation” refers to the excitatory cathodal electrode; the other side was given inhibitory anodal stimulation simultaneously.

As known extensively throughout GVS literature, subjects showed a wide range of tolerable and effective stimulus amplitudes, depending on stimulus length and quality of skin preparation. Subjects were presented serial stimulation of 3 ms rectangular pulses spaced 300 ms apart with increasing amplitudes to determine the highest tolerable amplitude over durations of 3–5 min. Stimulus amplitude was on average 1.8 mA (range 1–3 mA). Stimuli were presented in a randomized side order and inter-stimulus interval 300–500 ms, delivered in blocks of 3 min with 30 s pauses in between.

Short-duration GVS established for galvanic vestibular-evoked myogenic potentials (VEMPs) typically do not evoke a body tilting sensation, which was evoked at the individual’s specific amplitude and 300 ms pulse duration in all individual subjects, indicating toward a functionally sufficient amplitude.

### Galvanic Cervical VEMPs

One Ag/AgCl ring electrode was placed between upper and middle third of the sternocleidomastoid muscle on each side and each referenced against one on the upper sternum. EMG was recorded at a 10 μV/500 Hz range with software filters set DC to 25 kHz. Subjects were positioned in a chair with the rake flexed back to 45°. Subjects lifted their heads up from the back of the chair (39, 40) to activate the sternocleidomastoid muscles (41, 42). Eyes were closed (43) and the jaw slightly opened with closed mouth to minimize oculomotor, blink and masticatory artifacts, identical to our previous study setup. 500 stimuli were presented to each side.

### Galvanic Vestibular Cortical-Evoked Potentials

Subjects sat in relaxed position with the head leaned straight (40) against the back of the chair; closed eyes (39, 43) and opened jaw with closed mouth like in the VEMP recording, to minimize oculomotor, blink and masticatory EEG artifacts. 2,000 stimuli were presented to each side.

### EEG Recording

Every subject wore a head-size-fitted 10-20-EEG BrainCap with a total of 32 Ag/AgCl ring electrodes, including a right-sided infraorbital EOG channel. The cap was strapped to the chest by a Velcro harness, avoiding reflective masticatory activity compared to a chin strap fixation technique. Skin preparation was identical to the stimulation electrodes with the same chloride-free gel against capacitive effects and impedances less than 10 kΩ were achieved. EEG recordings were performed without hardware filters (DC with open high pass) at an amplitude resolution of 0.1 µV and 5,000 Hz sampling rate.

### Brain-Evoked Source Analysis (BESA)

For the analysis of possible intracranial sources of the recorded potentials (discrete dipole source analysis), BESA Research 5.3.7 with a Matlab interface was used. Data were software band pass filtered 0.1 Hz (12 dB) to 200 Hz (12 dB) with an additional 50 Hz notch filter of 1 Hz width. Segmentation and averaging by stimulus type and side were done for 0–180 ms post-stimulus. The predominating galvanic pulse artifact was identified by principal component analysis (PCA) and excluded by subsuming them into one source dipole (Figure 1); the galvanic pulse artifact presented as a rapidly decaying logarithmic curve outweighing the signal considerably within the first 20 ms. Following current BESA guidelines, two approaches to attribute potentials to brain regions were performed sequentially to the grand average of the 14 subjects, separately for left- and right-sided stimulation, analog to our previous study:

– *Sequential source seeding*: one dipole source was added to the model (“seeded”) after the other; their positions were sequentially computer-optimized, which minimized the error between data and model, until adding additional sources no longer could reduce the residual variance (RV equals unexplained data plus noise). No additional spatial seeding constraints were given.
– *Anatomical source seeding*: a total of five dipole sources (two symmetric pairs and one midline source) were placed in regions previously defined active under acoustic vestibular stimulation in our previous study and in accordance with imaging and *in vivo* experimental studies of the vestibular cortical network (3, 44, 45). These were the bilateral posterior parietal opercula (thereafter RPO/LPO for right/left parietal operculum) and the junction of anterior insula with inferior frontal gyrus [right anterior insula (RAI)/left anterior insula (LAI)]. An additional median regional source (frontal vestibular FV source) was placed fronto-dorsally to subsume long-latency awareness potentials and nearby bilateral areas 2v, 3aNV, and frontal eye fields (FEF). The regularization constant (RC) of the BESA model was varied between 0 and 20% upon adding each source to optimize source position at a RC = 1%, intended to minimize potential misplacement of sources by current source density crosstalk (BESA guidelines). All sources were spaced at least 3 cm apart, given the generally assumed 2 cm spatial resolution of discrete source analysis and to avoid major source crosstalk (BESA guidelines). The final positions of sources were further optimized by the computer within the millimeter range.

To model the active regions, three-dimensional regional sources were preferred over single dipole models, to subsume potentials in angular cortical structures into a single source of origin and accounting for the complex spatial arrangement of cortex layers in the peri-insular region. The cranial impedance model was the “adult, cr80” approximation supplied by BESA. This procedure for both approaches was then repeated with a basic four-shell ellipsoidal impedance model and without any software band pass and notch filters to rule out potential distortion of data.

The common best fit of regional sources from sequential and anatomical approaches of the grand averaged, software-filtered data was compared to the individual sequential and anatomical seeding models of each of the 14 subjects, one by one (34 models). Deviation of sources from the grand averaged data was considered negligible if they were located within 2 cm in normalized Talairach coordinates compared to the grand average model, given the assumed 2 cm spatial resolution of BESA. Activity peaks in active regions were considered for further analysis when they exceeded 2× baseline noise. Their latency relative to stimulus in msec and their amplitude in nano-Ampere meters (nAm) were recorded for statistical analysis. Over- and under-definition of the dataset with five regional sources were excluded by analysis in analogy to the previous study, probing with a varying RC between 1 and 20% and excess seeding of regional sources 3 cm apart from one another.

### Statistical Analysis

Statistical evaluation was performed with SPSS statistical software (SPSS20, IBM Corporation, Armonk, NY, USA) with Student’s two-sided *t*-test for comparison between peak latencies and amplitudes between regional sources. Furthermore, galvanic stimulation data were compared to acoustic vestibular stimulation data recorded in our previous study in the respective 14 subjects. An α-error *p* < 0.05 was considered statistically significant (marked *), *p* < 0.001 highly significant (**). ANOVA could not be performed since not all regions showed activity in a given subject at all intervals, analog to the previous study.

## Results

### Galvanic VEMP Analysis

EMG data were filtered with a band rejection filter at 50 Hz with a second-order slope. Stimuli were segmented and averaged for every side and stimulus (interval 0–40 ms). Peaks were marked (p13 largest positivity between 8 and 18 ms after mid-stimulus, n24 largest negativity between 18 and 24 ms) and relative amplitude noted. All subjects showed discernible ipsilateral p13–n24 amplitudes (in grand average 28.7 µA for right, 43.6 µA for left GVS; individual range 30–200 µA) in accordance with results by other authors (34–38). Further evaluation of galvanic VEMPs was not performed due to extensively rich data in the field.

### Cortical Regional Sources

After subtraction of the GVS artifact identified by PCA, the RV in grand average and in individual datasets was between 1 and 3%. The consensus model of sequential and anatomical source placement positioned a total of five sources in both anterior insular regions and posterior opercula and in a dorsal frontal region as summarized in Table 1, closely resembling the cortical sources of our previous acoustic stimulation study within less than 5 mm.

**Table 1:** Talairach consensus coordinates obtained by sequential and anatomical seeding of regional sources in brain-evoked source analysis (BESA) for galvanic vestibular stimulation in comparison to those obtained in acoustic vestibular stimulation in the previous study.

	Normalized Talairach coordinates (mm)					
	Galvanic vestibular stimulation			Acoustic vestibular stimulation		
	*x*	*y*	*z*	*x*	*y*	*z*
FV	±0	26	52	±0	24	51
RAI/LAI	±46	28	−2	±47	27	2
RPO/LPO	±31	−36	19	±31	−40	22

*Modeled corresponding regions were located well within the 20 mm spatial accuracy of the BESA method (no deviation >5 mm in any orthogonal direction). Scale in millimeters*.

### Galvanic-Specific and Common Patterns of Activation

In galvanic stimulation, subjects showed regional source activities in the anterior insular RAI/LAI and posterior opercular regions RPO/LPO at 25, 35, 50, 80, and 110 ms post-stimulus; in the frontal region at 35, 45, 70, and 110 ms. Not every single subject showed activity in all regions at all possible intervals, but at least two regions were consistently active at each possible time interval in lateral regions and in three of four possible frontal activity periods. This pattern of activation made analysis with ANOVA not feasible; therefore, differences in latencies and amplitudes were investigated with two-sided *t*-tests (see Methods), analog to our previous study.

**Figure 2:**
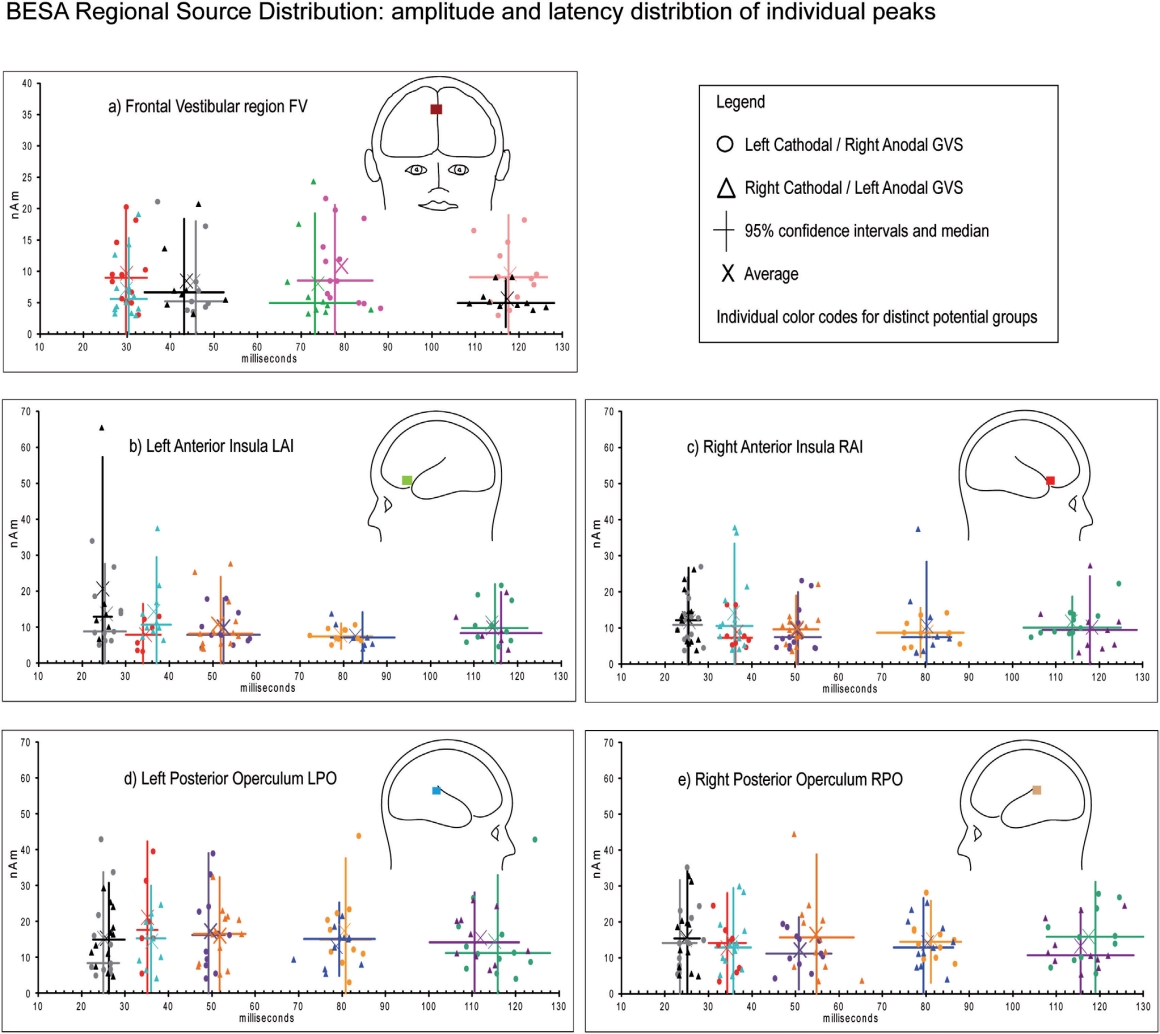
Galvanic vestibular-evoked potentials in the five investigated cortical regions FV **(A)**, right anterior insula (RAI) **(B)**, left anterior insula (LAI) **(C)**, right posterior operculum (RPO) **(D)**, and left posterior operculum (LPO) **(E)** with the individual subjects’ potential latencies and amplitudes for left cathodal stimulation (circles) and right stimulation (triangles) with individual colors to distinguish potential clusters at 25, 35, 50, 80, and 110 ms in RAI, LAI, RPO, and LPO and 30, 45, 70, and 110 ms in frontal regions FV. The potential latencies were significantly different from one another within one region (Table 2), whereas no explicit effects were found for side of stimulation on either amplitude or latency or between cortical regions of interest.

Figure 1 shows the time course of regional source dipoles in a grand averaged model of all 14 subjects for left-sided stimulation. Figure 2 demonstrates the individual and average evoked potential amplitudes and latencies of left and right galvanic stimulation. All potential periods in a respective region were distinct in latency (virtually all *p* < 0.001, Table 2) from one another. There was no effect of left/right side of stimulation on the latency or amplitude in any region or time period.

**Table 2:** Comparison of evoked potentials for regional sources right anterior insula (RAI) and left anterior insula (LAI), right posterior operculum (RPO) and left posterior operculum (LPO) and frontal regions (FV) for bipolar galvanic stimulation with cathode left (LE) or right (RI) at time intervals 25, 35, 50, 80, and 110 for RAI, LAI, RPO and LPO regions and 30, 45, 70, and 110 ms for frontal regions.

Stimulus side (LE or RI)			Regional Source				

	Latency		LAI	LPO	RAI	RPO	FV
LE	25–35		***p* = 0.002**	***p* < 0.001**	***p* < 0.001**	***p* < 0.001**	
	35–50		***p* < 0.001**	***p* < 0.001**	***p* < 0.001**	***p* = 0.004**	
	50–80		***p* < 0.001**	***p* < 0.001**	***p* < 0.001**	***p* < 0.001**	
	80–110		***p* < 0.001**	***p* < 0.001**	***p* < 0.001**	***p* < 0.001**	
	30–45						***p* < 0.001**
	45–70						***p* < 0.001**
	70–110						***p* < 0.001**

RI	25–35		***p* < 0.001**	***p* < 0.001**	***p* < 0.001**	***p* < 0.001**	
	35–50		***p* < 0.001**	***p* < 0.001**	***p* < 0.001**	***p* < 0.001**	
	50–80		***p* < 0.001**	***p* < 0.001**	***p* < 0.001**	***p* < 0.001**	
	80–110		***p* = 0.001**	***p* < 0.001**	***p* < 0.001**	***p* < 0.001**	
	30–70						***p* < 0.001**
	45–70						***p* < 0.001**
	70–110						***p* < 0.001**

LE–RI	25		*p* >> 0.05	*p* >> I0.05	*p* >> 0.05	*p* >> 0.05	
	30						*p* >> 0.05
	35		*p* >> 0.05	*p* >> 0.05	*p* >> 0.05	*p* >> 0.05	
	45						*p* >> 0.05
	50		*p* >> 0.05	*p* >> 0.05	*p* >> 0.05	*p* >> 0.05	
	70						***p* = 0.02 LE > RI**
	80		*p* >> 0.05	*p* >> 0.05	*p* >> 0.05	*p* >> 0.05	
	110		*p* >> 0.05	*p* >> 0.05	*p* >> 0.05	*p* >> 0.05	*p* >> 0.05

		Amplitude LE–RI	LAI	LPO	RAI	RPO	FV
		25	*p* >> 0.05	*p* >> 0.05	*p* >> 0.05	*p* >> 0.05	
		30					*p* >> 0.05
		35	*p* >> 0.05	*p* >> 0.05	***p* = 0.02 RI > LE**	*p* >> 0.05	
		45					*p* >> 0.05
		50	*p* >> 0.05	*p* >> 0.05	*p* >> 0.05	*p* >> 0.05	
		70					*p* >> 0.05
		80	*p* >> 0.05	*p* >> 0.05	*p* >> 0.05	*p* >> 0.05	
		110	*p* >> 0.05	*p* >> 0.05	*p* >> 0.05	*p* >> 0.05	***p* = 0.02 LE > RI**

LE amplitude at 25	LAI	LPO	RAI	RI amplitude at 25	LAI	LPO	RAI
LAI				LAI			
LPO	i >> 0.05			LPO	*p* >> 0.05		
RAI	*p* >> 0.05	*p* >> 0.05		RAI	*p* >> 0.05	*p* >> 0.05	
RPO	*p* >> 0.05	*p* >> 0.05	*p* >> 0.05	RPO	*p* >> 0.05	*p* >> 0.05	*p* >> 0.05

LE amplitude at 35	LAI	LPO	RAI	RI amplitude at 50	LAI	LPO	RAI
LAI				LAI			
LPO	*p* >> 0.05			LPO	*p* = 0.07		
RAI	*p* >> 0.05	*p* = 0.07		RAI	*p* >> 0.05	*p* >> 0.05	
RPO	*p* >> 0.05	*p* >> 0.05	*p* = 0.06	RPO	***p* = 0.01 RPO > LAI**	*p* >> 0.05	*p* >> 0.05

LE amplitude at 50	LAI	LPO	RAI	RI amplitude at 80	LAI	LPO	RAI
LAI				LAI			
LPO	***p* = 0.01 LPO > LAI**			LPO	*p* = 0.01		
RAI	*p* >> 0.05	***p* = 0.03 LPO > RAI**		RAI	*p* >> 0.05	*p* = 0.02	
RPO	*p* >> 0.05	***p* = 0.04 LPO > RPO**	***p* = 0.049 RPO > RAI**	RPO	*p* = 0.01	*p* >> 0.05	*p* = 0.07

LE amplitude at 80	LAI	LPO	RAI	RI amplitude at 80	LAI	LPO	RAI
LAI				LAI			
LPO	***p* = 0.02 LPO > LAI**			LPO	*p* >> 0.05		
RAI	*p* >> 0.05	***p* = 0.02 LPO > RAI**		RAI	*p* >> 0.05	*p* >> 0.05	
RPO	***p* = 0.001 RPO > LAI**	*p* >> 0.05	***p* = 0.02 RPO > RAI**	RPO	*p* >> 0.05	*p* >> 0.05	*p* >> 0.05

LE amplitude at 110	LAI	LPO	RAI	RI amplitude at 110	LAI	LPO	RAI
LAI				LAI			
LPO	*p* >> 0.05			LPO	***p* = 0.01 LPO > LAI**		
RAI	*p* >> 0.05	*p* >> 0.05		RAI	***p* = 0.04 RAI > LAI**	***p* = 0.01 LPO > RAI**	
RPO	***p* = 0.01 RPO > LAI**	*p* >> 0.05	***p* = 0.04 RPO > RAI**	RPO	*p* >> 0.05	*p* >> 0.05	*p* >> 0.05

*All potentials are significantly different in latency defining them as individual entities with no major differences in potential amplitudes between left and right or between sides of stimulation*.

*Statistically significant p values are highlighted in bold print*.

In comparison to the activations by acoustic stimulation in our previous study (activation in RAI, LAI, RPO, and LPO at 20, 30, 60, and 110 ms; in frontal regions at 70 and 110 ms), galvanic activations were significantly different from their acoustic counterparts at 25, 35, 50, and 80 versus 20, 30, and 60 ms in RAI, LAI, RPO, and LPO and the early frontal galvanic activations at 30 and 45 ms had no respective counterparts in acoustic stimulation. Activation periods in common with galvanic and acoustic stimulation were the 70 ms in frontal regions and the 110 ms period in all lateral and frontal regions (Table 3). However, even in potentials with the same latency shared by galvanic and acoustic stimulation, the direction of the regional source dipole was differently oriented in space (compare Figure 1 of this study with Figure 3 of Ref. (8), see Discussion).

**Table 3:** Comparison between potentials evoked by galvanic stimulation and acoustic stimulation from the previous study (8).

Latency differences				Frontal	Acoustic	70 ms	110 ms				

Galvanic vs. acoustic vestibular stimulation				Galvanic							
				30 ms							
		Left side stimulus		45 ms							
				70 ms		*p* = 0.05					
				110 ms			*p* >> 0.05				

**Left anterior insula (LAI)**	Acoustic	20 ms	30 ms	60 ms	110 ms	**Right anterior insula (RAI)**	Acoustic	20 ms	30 ms	60 ms	110 ms
Galvanic						Galvanic					
25 ms		***p* < 0.001**	***p* < 0.001**			25 ms		***p* < 0.001**	***p* < 0.001**		
35 ms		***p* = 0.001**	*p* >> 0.05			35 ms		***p* < 0.001**	***p* = 0.005**		
50 ms				***p* = 0.009**		50 ms				***p* < 0.001**	
80 ms				***p* = 0.003**		80 ms				***p* = 0.001**	
110 ms					*p* >> 0.05	110 ms					*p* >> 0.05

**Left posterior operculum (LPO)**	Acoustic	20 ms	30 ms	60 ms	110 ms	**Right posterior operculum (RPO)**	Acoustic	20 ms	30 ms	60 ms	110 ms
Galvanic						Galvanic					
25 ms		*p* = 0.06	***p* < 0.001**			25 ms		***p* = 0.008**	***p* < 0.001**		
35 ms		***p* = 0.04**	*p* = 0.05			35 ms		***p* < 0.001**	*p* >> 0.05		
50 ms				***p* = 0.001**		50 ms				***p* = 0.04**	
80 ms				***p* = 0.005**		80 ms				***p* = 0.01**	
110 ms					*p* >> 0.05	110 ms					*p* = 0.06
				**Frontal**	Acoustic	70 ms	110 ms				
				Galvanic							
				30 ms							
		Right side stimulus		45 ms							
				70 ms		*p* >> 0.05					
				110 ms			*p* >> 0.05				

**LAI**	Acoustic	20 ms	30 ms	60 ms	110 ms	**RAI**	Acoustic	20 ms	30 ms	60 ms	110 ms
Galvanic						Galvanic					
25 ms		*p* = 0.05	*p* >> 0.05			25 ms		***p* = 0.005**	***p* < 0.001**		
35 ms		***p* = 0.001**	***p* = 0.006**			35 ms		***p* < 0.001**	***p* = 0.04**		
50 ms				***p* = 0.01**		50 ms				***p* = 0.03**	
80 ms				***p* = 0.003**		80 ms				***p* = 0.01**	
110 ms					*p* >> 0.05	110 ms					***p* = 0.03**

**LPO**	Acoustic	20 ms	30 ms	60 ms	110 ms	**RPO**	Acoustic	20 ms	30 ms	60 ms	110 ms
Galvanic						Galvanic					
25 ms		***p* = 0.01**	***p* < 0.001**			25 ms		***p* = 0.02**	***p* = 0.003**		
35 ms		***p* = 0.001**	***p* = 0.03**			35 ms		***p* < 0.001**	***p* = 0.04**		
50 ms				***p* < 0.001**		50 ms				*p* >> 0.05	
80 ms				***p* = 0.003**		80 ms				***p* = 0.005**	
110 ms					*p* >> 0.05	110 ms					*p* >> 0.05

*Galvanic-evoked potentials in right and left anterior insula (RAI and LAI), right and left posterior operculum (RPO and LPO) and frontal regions (FV) for bipolar galvanic stimulation with cathode left (LE) or right (RI) at time intervals 25, 35, 50, 80 and 110 for RAI, LAI, RPO and LPO regions and 30, 45, 70 and 110 ms for frontal regions. Acoustic potentials from the previous study occurred at 20, 30, 60 and 110 ms in regions RAI, LAI, RPO and LPO and at 70 and 110 ms in FV*.

*The galvanic 25, 35, 50 and 80 ms potentials in RAI, LAI, RPO and LPO are shown to be distinct entities by significant latency differences for both left and right galvanic or acoustic stimulation. No significantly different latencies were found for 70 ms in FV and in all regions at 110 ms, indicating common entities. The 30 and 45 ms potentials in FV under galvanic stimulation had no equivalents in acoustic vestibular stimulation*.

*Statistically significant p values are highlighted in bold print*.

## Discussion

The multifocal, bilateral, and multisensory vestibular network was investigated with GVS directed specifically at the semicircular canal organs for activation latencies and electroencephalographic peak amplitudes in comparison to our previous study, which used specific acoustic stimuli directed at the sacculus otolith organ beyond the electrophysiologically well-investigated 20 ms period. This vestibular network has previously been probed thoroughly in anatomical, functional metabolic, and invasive stimulation studies.

Discrete source analysis of electric dipoles was used to localize the origin and time course of these potentials, revealing galvanic-specific medium latency potentials (25–80 ms) in contrast to long-latency potentials (70–110 ms) in common with acoustic otolith-evoked stimulation of the previous study.

### Multimodality and the Vestibular Cortical Regions

The peripheral vestibular organ is comprised of three semicircular canals for angular acceleration measurement, which are particularly susceptible to GVS and the two otolith organs utriculus and sacculus, of which the sacculus for vertical translational motion detection can be selectively stimulated with very specific acoustic stimuli (8, 9, 46).

Differential processing of vestibular information from semicircular canals and otoliths has already been described at the level of the vestibular nuclei (47) up to the thalamus (48); however, there is only scarce data on differences of rotational versus translational sensory input processing on a cortical level, studies on which are centered around top-down concepts of directionality versus spatial reference frames [review in Ref. (49, 50)]. An electrophysiological bottom-up analysis of differential cortical effects at a cortical level between semicircular canal versus otolith stimulation has not been performed to date. In this study, short-duration GVS with proven vestibulo-spinal gVEMP effect was used.

After the first 20 ms after a sensory cue, primary vestibular information has ascended through the peripheral vestibular nerve and vestibular nuclei up through thalamus, likely into early activated vestibular cortical patches. Prior to the thalamus, well-studied phenomena like oculomotor-related oVEMPs or vestibulo-spinal reflexes like sternocleidomastoid VEMP are elicited, on which there is extensive literature and reviews elsewhere (4, 5, 7, 39, 51–59).

In the vestibular cortical network multiple regions around an inner vestibular core including the bilateral anterior insula (RAI, LAI) and posterior operculum (RPO, LPO) engage in mutual crosstalk of higher-level sensory data. They integrate vestibular, proprioceptive, visual, and possibly other cues (such as higher-order sensory inputs or re-afference from vestibular-related reflexes) into an overall concept of body position and motion in space [reviewed in Ref. (1–3, 46, 49, 50, 60)].

### Galvanic-Evoked Cortical Vestibular Potentials

Binaural bipolar GVS known to stimulate the semicircular canals eliciting a sensation of whole-body-tilt to the cathodal side [(61) and references therein] was able to evoke vestibular reflex neck muscle responses as gVEMPs in all of the investigated subjects, reflecting intact connectivity of peripheral afferent and descending efferent vestibular tracts. This effect also emphasizes the vestibular system influence of short-term (<10 ms) GVS in comparison to long-term stimulation of several 100 ms duration. Beyond the well-studied initial 20 ms post-stimulus period, sets of distinctively separate potential peaks could be isolated in regions of vestibular network denomination: bilateral anterior insula, posterior operculum, and a frontal region, which was modeled to subsume widespread frontal activity including areas 2v, 3nv, and FEF. Potentials in RAI/LAI and RPO/LPO showed identical latencies among each other, whereas the frontal regions exhibited distinctively earlier patterns of activation, when compared to acoustic stimulation.

### Differential and Common Effects of Semicircular Canal and Otolith Stimulation

Comparison of peak latencies between currently studied galvanic-evoked and acoustic-evoked vestibular potentials of our previous study revealed that the intermediate latency galvanic potentials at 25, 35, 50, and 80 ms in the lateral regions RAI/LAI/RPO/LPO were distinctively different from their acoustic-evoked counterparts at 20, 30, and 60 ms. In the frontal regional source galvanic-evoked potentials at 30 and 45 ms could be demonstrated, which had no acoustic-evoked counterpart, indicating different pathways for possibly semicircular canal-related angular acceleration input versus translational motion in higher-order centers.

Late potentials at 70 ms in FV and 110 ms in all of the investigated regions were found at no statistically significant latencies between galvanic and acoustic vestibular stimulation [Figure 1 of this study compared to Figures 3 and 4 of Ref. (8)], indicating a common pattern of multimodal signal post-processing in these frontal regions. This might indicate aforementioned multisensory integration in these centers. Among the evoked potentials with the same latencies within one region, there were notably different lead dipole orientations. This might indicate that within a given cortical region, different cortical patches possibly tasked with different peripheral vestibular qualities and with a different spatial orientation toward the skull surface may be activated. This grand-scale effect however does not allow drawing conclusions about individual neuron response characteristics within these regions to specific peripheral stimuli. These effects relate to different metabolic activation patterns in fMRI and PET studies during different vestibular stimulation modalities.

### Vestibular versus Somatosensory Co-Stimulation

Galvanic vestibular stimulation includes sensory afferent components of cutaneous nerve fibers, especially unmyelinated pain fibers, depending on stimulus parameters. Particularly the intermediate latency P60 and N80 somatosensory-evoked potentials (SEPs) over the contralateral primary somatosensory cortex S1 could have contributed to activity in the parietal source dipoles at the respective intervals, especially since the S1 face/neck area is positioned near the 2 cm source crosstalk boundary of the parietal RPO/LPO region (see Methods). A somatosensory contribution particularly to the 80 ms RPO/LPO activation is therefore likely. However, the contribution to the RPO/LPO potentials is likely small, since only cathodal stimulation evokes considerable discomforting sensations and there was no significant difference in potential amplitudes for the side of cathodal stimulation. The anterior RAI/LAI dipoles at the respective intervals show exclusively vestibular-related activation because of the distance to S1 with its activations. Comparative sham stimulation with the given stimulus parameters away from the mastoid bones was discarded at early stages of the study, because source interference of SEP with the posterior dipoles was inherently unavoidable and neither would the different orientation of the stimulus dipole have aided in differential artifact removal.

### Possible Clinical Impact

Despite its long historical track record, GVS has traditionally been a research tool scarcely used in routine clinical electrophysiology. Galvanic stimulators for patient use are typically prohibitively expensive and used for one single task. Tools like acoustic vestibular stimulation, as demonstrated in the previous study, can be more readily implemented to practical application with commercially available clinical electrophysiology devices, as demonstrated for Laplacian montages in an 8-channel EMG device for acoustic vestibular cortical potentials. There is however evidence on temporary acoustic threshold shifts as a noxious inner ear effect, even with low numbers of acoustic VEMP (62), implying the possibility of permanent hearing damage with the number of iterations required for cortical potentials versus VEMP in typically older patients with possible pre-existing hearing deficits. In contrast, there are no known permanent side effects of GVS.

To establish galvanic cortical potentials as a clinically available technique, it will require simplified galvanic stimulator hardware with automated stimulus presentation in conjunction with a clinical EEG device running a simplified and user-friendly BESA routine to be used by clinical technicians.

Certain peripheral vestibular disorders preferentially affect some parts of the vestibular organs while mostly sparing the other and in some cases the function of specific vestibular organs may indicate the further course of the disease. Examples of which are vestibular neuritis or Meniere’s disease (63, 64). How these disorders affect evoked potentials of the vestibular cortical system and how possible changes imply or correlate with different clinical outcomes needs to be investigated further. Testing patients with the respective diseases repeatedly during restitution or between relapses with both techniques might show correlation between specific potential alterations in one or both tests in conjunction with the clinical course.

## Conclusion

Galvanic vestibular stimulation of the semicircular canal nerves was shown to evoke specific potentials in known multimodal vestibular cortical regions. Intermediate latency potentials ranging from 25 to 80 ms were found at latencies proprietary to semicircular canal input in comparison to acoustic-evoked sacculus-related potentials (20–60 ms) on the same time frame. Late potentials (70–110 ms) were revealed to be at identical latencies between semicircular (galvanic) and otolith (acoustic) stimulation. Dipole orientation indicated the activation of different cortical patches within given cortical regions. It was demonstrated that these different peripheral vestibular afference modalities had different cortical response patterns in a bottom-up approach, whereas previous anatomical, physiological, and functional imaging studies had pointed out multimodal higher-order spatial reference frame and motion in space concepts in a top-down approach.

Certain peripheral vestibular disorders affect semicircular canals and otoliths differently. Their effect on a cortical post-processing level may be the focus of further research and potentially eventual clinical application.

## Ethics Statement

This study protocol was carried out in accordance with recommendations of decision 142/04 of the LMU ethics committee with written informed consent from all subjects in accordance with the Declaration of Helsinki.

## Author Contributions

SK and KB designed the study. SK and AS developed the setup and performed the experiments and data analysis. SK and KB composed the manuscript.

## Conflict of Interest Statement

The authors declare that the research was conducted in the absence of any commercial or financial relationships that could be construed as a potential conflict of interest.
